# Decomposing low birth weight inequality in Lesotho: A distributional analysis using hybrid Fairlie and recentred influence function methods on multiple indicator cluster survey 2018

**DOI:** 10.4102/jphia.v17i1.1603

**Published:** 2026-04-23

**Authors:** Topollo E. Motlamelle, Ratjomose P. Machema

**Affiliations:** 1 Social Impact Division, MOPSY Group, Centurion, South Africa; 2 Department of Economics, Faculty of Social Sciences, National University of Lesotho, Maseru, Lesotho

**Keywords:** low birth weight, inequality, RIF regression, Fairlie decomposition, Lesotho

## Abstract

**Background:**

Low birth weight (LBW) is a major public health concern in Lesotho, linked to higher infant mortality, long-term morbidity and intergenerational poverty. Despite expanded maternal and child health services, socio-economic disparities in birth outcomes persist.

**Aim:**

This study investigates the drivers of LBW inequality, quantifying and decomposing socio-economic and geographic disparities to inform equity-oriented health policy.

**Setting:**

The study used nationally representative data from the 2018 Lesotho Multiple Indicator Cluster Survey, covering all 10 districts.

**Methods:**

A hybrid econometric framework was applied. Low birth weight inequality was first assessed by using the Concentration Index (CI), Erreygers Index (EI) and Wagstaff Index (WI). Fairlie decomposition was then used to compare LBW probabilities between poor vs. rich and rural vs. urban households. Finally, recentred influence function regressions examined the distributional impact of maternal and household factors across the 28th, 50th and 75th birth weight percentiles.

**Results:**

Maternal education, antenatal care (ANC) attendance, maternal disability and digital connectivity were key determinants of LBW, with effects varying across the birth weight distribution. Only 20.5% of the poor–rich LBW gap was explained by observable characteristics, primarily education and ANC. Recentred influence function regressions revealed stronger protective effects of education and ANC at lower percentiles, while maternal disability and Internet access were more relevant at higher percentiles.

**Conclusion:**

LBW in Lesotho reflects both structural barriers and compositional disadvantages. Addressing these requires equity-focused, distribution-sensitive maternal health interventions.

**Contribution:**

This study provides evidence-based insights to inform targeted policies to reduce LBW among vulnerable populations.

## Introduction

Low birth weight (LBW), defined by the World Health Organization (WHO) as birth weight less than 2500 g, is a key early-life health challenge in low- and middle-income countries.^1^ Beyond its clinical relevance, LBW is widely recognised as a powerful indicator of social disadvantage. Low birth weight is strongly associated with neonatal and infant mortality, developmental impairments, cognitive delays and heightened vulnerability to non-communicable diseases later in life.^2,3^ Importantly, LBW also contributes to intergenerational transmission of poverty, as children born with LBW are more likely to experience educational setbacks and reduced economic productivity in adulthood.^2,4^ As such, LBW remains one of the most persistent indicators of early-life inequality and is increasingly used to monitor progress towards global health equity targets.

Globally, LBW prevalence is estimated at 14.6%,^3^ with sub-Saharan Africa (SSA) reporting some of the highest rates. Lesotho reflects these regional trends, with recent estimates indicating that LBW remains a significant public health concern despite sustained investments in maternal and child health services.^4,5^ The persistence of LBW in this context highlights that improvements in health system coverage alone may be insufficient to address underlying disparities. In low-income settings such as Lesotho, LBW therefore represents not only a public health challenge but also a broader development concern.

Empirical evidence consistently shows that LBW is unequally distributed, with a greater burden borne by children from poor households, rural communities and mothers with limited access to healthcare and education.^6,7,8,9^ These disparities reflect deeper structural inequalities embedded within Lesotho’s health, social and economic systems.^10,11^ Addressing LBW in such contexts, therefore, requires moving beyond biomedical explanations to examine the socio-economic gradients shaping early-life outcomes.

The literature identifies a wide range of maternal and household-level factors associated with LBW across SSA. At the maternal level, age, parity, education, nutritional status, disability status and antenatal care (ANC) utilisation are repeatedly shown to influence birth outcomes.^10,12,13^ Household characteristics – including income, sanitation conditions, food security and access to information and services – further shape maternal health and care-seeking behaviours, thereby indirectly affecting birth weight.^14^ Regional studies also report systematic differences by child sex, place of residence and maternal disability, underscoring the role of spatial and demographic heterogeneity in shaping LBW risk.^9,11^ Collectively, these findings point to persistent socio-spatial inequalities in access to healthcare and foundational infrastructure.

Among these determinants, maternal education consistently emerges as one of the most protective factors. Specifically, educated mothers are more likely to access health services, comply with medical guidance and adopt positive health behaviours during pregnancy. However, in Lesotho, persistent disparities in education and healthcare access continue to translate into unequal birth outcomes. Nwako, Mashego and Ndinda, in their 2020 study, *using Demographic and Health Survey (DHS)* data from multiple Southern African countries, found that maternal education and wealth are primary LBW drivers, with ANC attendance acting as a key mediating factor.^9^ Their findings underscore the importance of decomposition approaches that can disentangle not only the magnitude of inequality but also its underlying structure – an analytical gap this study seeks to address in the Lesotho context.

While decomposition techniques have been widely applied to health outcomes, such as in nutrition,^15^ immunisation^7^ and maternal healthcare utilisation,^16^ relatively few studies integrate rank-dependent inequality indices with both binary and distribution-sensitive decomposition methods, particularly in low-income settings and in analyses of LBW.^17^ Moreover, existing studies on Lesotho remain limited in their ability to jointly examine incidence-based disparities and distributional dynamics of birth weight.

Against this background, this study investigates inequality in LBW outcomes in Lesotho, using nationally representative data from the 2018 Multiple Indicator Cluster Survey (MICS). This study contributes to the literature in two key ways. Firstly, it deploys a hybrid econometric framework that integrates both incidence-based inequality and distribution-sensitive approaches.^18,19^ Secondly, it generates policy-relevant evidence to inform more equitable maternal and child health interventions. To the best of the authors’ knowledge, no prior study has examined LBW inequality in Lesotho, using an integrated decomposition framework that explicitly models locality. As such, this study advances the empirical basis for equity-oriented policy design and contributes directly to the monitoring of Sustainable Development Goals (SDG) 3 and 10.

Consistent with the equity-monitoring objectives of the 2018 Lesotho MICS, this study is guided by three specific analytical objectives: (1) to measure the extent of socio-economic inequality in low birth weight among children under 5, (2) to decompose the observed inequality between poor–rich and rural–urban groups, using hybrid econometric methods, and (3) to examine how maternal and household characteristics influence the entire birth weight distribution. Accordingly, the study addresses the following research questions: how unequal are LBW outcomes in Lesotho? What factors explain these inequalities? And do these factors vary across the birth weight distribution?

Consistent with established practice, LBW is defined here as ≤ 2.5 kg, to address potential heaping at the 2.5-kg threshold common in household survey data.^20^ Using a combination of concentration indices – Concentration Index (CI), Erreygers Index (EI) and Wagstaff Index (WI) – alongside decomposition techniques (Fairlie, Recentred Influence Function [RIF] and Non-Linear Blinder-Oaxaca [NLD]), the analysis captures both observable and structural drivers of LBW inequality. This dual approach allows for a nuanced assessment of how socio-economic disparities in LBW manifest across population subgroups and along the full birth weight distribution.

## Research methods and design

### Study design

This study adopts a hybrid econometric design, integrating rank-dependent indices, non-linear decompositions and distribution-sensitive regressions to analyse LBW disparities in Lesotho. Given the non-linear nature of LBW determinants, these decomposition approaches allow attribution of observed disparities to measurable covariates and unobserved factors.

### Setting

The study uses the 2018 Lesotho Multiple Indicator Cluster Survey (MICS6), a nationally representative household survey conducted across all 10 districts. The MICS programme was designed to support equity-oriented monitoring of maternal and child health outcomes, with particular emphasis on identifying socio-economic gradients and population subgroups at risk. Within this framework, birth weight serves not only as a clinical indicator but also as a tracer of cumulative maternal, household and structural disadvantage.

### Study population and sampling strategy

The analytic sample includes 731 children under 5 years of age and their mothers aged 15–49 years, drawn from merged household and women’s datasets. Of the initial 850 women who reported a child’s birth weight in the merged dataset, observations were excluded if the respondent did not know the birth weight or did not report the number of antenatal care visits. After these exclusions, the final analytic sample comprised 731 mother–child pairs, representing 14% of the initial sample being dropped as a result of missing key variables. These variables were required for the Fairlie and RIF decompositions and could not be imputed reliably.

A complete-case approach was applied to manage missing data. Coefficients from preliminary models using the full sample (including cases with imputed or missing ANC and birth weight) were compared with those from the final restricted sample to assess the potential sensitivity to this decision. No substantive differences in direction or magnitude were observed, suggesting that the exclusion of incomplete cases did not materially affect the results.

### Data collection

Data for this study were obtained from the 2018 Round 6 of the MICS, implemented by the Lesotho Bureau of Statistics with technical support from UNICEF. The Multiple Indicator Cluster Survey employs a stratified two-stage sampling design covering all 10 districts of Lesotho and is widely used for equity-oriented monitoring of maternal and child health outcomes.

This study uses secondary, anonymised microdata from the publicly available MICS datasets. Women’s and household files were merged by using unique identifiers. The analytic sample was restricted to women aged 15–49 with complete and valid information on birth weight and antenatal care utilisation. Observations with missing or implausible values on key analytical variables were excluded in line with the complete-case approach described in the previous section.

Data quality is assured within the MICS framework through standardised survey instruments, enumerator training, field supervision and electronic data capture with built-in validation checks. No additional data collection was undertaken for this study beyond cleaning, merging and variable construction in accordance with MICS guidelines.

### Measurement of variables

Low birth weight served as the primary outcome variable. It was operationalised as a binary indicator equal to one, if the reported birth weight was less than or equal to 2.5 kg, and zero otherwise, consistent with the WHO guidelines and adjusted to account for heaping at the threshold commonly observed in household survey data.^20^ For distributional analyses, birth weight was additionally treated as a continuous variable measured in kilograms.

Maternal characteristics included age (measured in completed years), educational attainment, marital status, functional disability status, ANC utilisation and tetanus toxoid injections. Maternal education was categorised into three mutually exclusive groups: no or primary education, secondary education and higher education. Marital status was coded into standard MICS categories (married, widowed, divorced, separated and never married). Functional disability was measured by using the MICS functional difficulty module and coded as a binary indicator distinguishing mothers reporting no functional difficulty from those reporting at least one functional limitation. Antenatal care utilisation was measured as the total number of ANC visits attended during pregnancy, treated as a continuous variable. Tetanus toxoid exposure was measured as the number of injections received during pregnancy.

Child characteristics included sex, coded as a binary variable (girls = 1, boys = 0).

Household-level variables captured socio-economic and infrastructural conditions. Household wealth was measured by using the MICS wealth index, constructed via a principal component analysis of household assets and amenities. Both continuous wealth score and categorical wealth quintiles were used, with quintiles grouped where required for binary decompositions. Residential locality was coded as a binary indicator distinguishing rural from urban households. Household size was measured as the total number of household members.

Access to digital connectivity was measured by using a binary indicator of household Internet access. Environmental variables included the source of drinking water, toilet facility type and primary household energy source, each categorised according to standard MICS classifications and recoded into analytically meaningful groups. All variables (as seen in Table 1^21^) were harmonised to ensure consistent measurement across inequality indices, decomposition analyses and distribution-sensitive regressions.

**TABLE 1:** Summary statistics of key variables.

Variables	Obs	Mean	s.d.	Min	Max
**Outcome variable(s)**					
Child birth weight (*continuous*)	731	3.015	0.589	0.6	5.6
Low birth weight (1 ≤ 2.5 kg, 0 = otherwise)	731	0.216	0.412	0.0	1.0
***Explanatory variables by category:***					
**Maternal & Child characteristics**					
Maternal age	731	25.886	6.136	15.0	46.0
Antenatal care visits	731	5.201	2.157	1.0	20.0
Tetanus toxoid injection doses	731	2.230	1.059	1.0	6.0
Maternal functional disability (1 = None, 0 = Present)	731	0.064	0.245	0.0	1.0
Locality (1 = Rural, 0 = Urban)	731	0.689	0.463	0.0	1.0
Child’s gender (1 = Female, 0 = Male)	731	0.487	0.500	0.0	1.0
Maternal education level:					
• Primary education or none	731	0.349	0.477	0.0	1.0
• Secondary education	731	0.568	0.496	0.0	1.0
• Higher education	731	0.083	0.277	0.0	1.0
Maternal marital status:					
• Married	731	0.781	0.414	0.0	1.0
• Widowed	731	0.029	0.167	0.0	1.0
• Divorced	731	0.012	0.110	0.0	1.0
• Separated	731	0.023	0.151	0.0	1.0
• Never married	731	0.155	0.362	0.0	1.0
**Household characteristics**					
Household size	731	5.174	2.293	1.0	18.0
Access to internet (1 = Yes, 0 = No)	731	0.354	0.479	0.0	1.0
Wealth index quintile:					
• Poorest	731	0.295	0.457	0.0	1.0
• Second	731	0.207	0.405	0.0	1.0
• Middle	731	0.190	0.393	0.0	1.0
• Fourth	731	0.189	0.392	0.0	1.0
• Richest	731	0.119	0.324	0.0	1.0
Type of water source:					
• Piped water	731	0.761	0.427	0.0	1.0
• Well water	731	0.070	0.255	0.0	1.0
• Spring water	731	0.159	0.366	0.0	1.0
• Tank water	731	0.003	0.052	0.0	1.0
• Surface water	731	0.008	0.090	0.0	1.0
Type of toilet facility:					
• Flush toilet	731	0.016	0.127	0.0	1.0
• Pit toilet	731	0.720	0.450	0.0	1.0
• None or bush or field	731	0.264	0.441	0.0	1.0
Type of energy source:					
• Paraffin	731	0.625	0.484	0.0	1.0
• Electricity	731	0.363	0.481	0.0	1.0
• Other sources like gas, biomass, etc.	731	0.012	0.110	0.0	1.0

*Source*: Lesotho Bureau of Statistics (BoS), United Nations Children’s Fund (UNICEF). Lesotho Multiple Indicator Cluster Survey 2018, Survey Findings Report. Maseru: BoS and UNICEF; 2019

Notes: *Obs* indicates the observations. Table 1 also reports the means, standard deviations (s.d.), minimum (min) values and maximum (max) values, respectively.

Internet access indicates whether mothers have access to digital information. Sample size *n* = 731 reflects complete data across all variables.

### Data analysis

The analytical strategy combined rank-dependent inequality measures, non-linear decomposition techniques and distribution-sensitive regression methods to examine the socio-economic disparities in LBW in Lesotho.

Specifically, associations between covariates and LBW were examined by using a multi-stage analytical strategy. Socio-economic inequality in LBW was quantified by using Rank-dependent measures, including the CI and its corrected forms, the EI and WI, with household wealth rank serving as the socio-economic ordering variable.^22^ These indices capture the direction and magnitude of inequality but do not identify the underlying drivers.

Fairlie decomposition was applied to the binary LBW outcome in order to decompose differences in predicted probabilities between poor–rich and rural–urban groups into explained and unexplained components, to attribute observed disparities to measurable characteristics. A non-linear Blinder-Oaxaca decomposition based on logistic regression was then used as a complementary approach to distinguish between differences arising from group characteristics (endowments) and differences in coefficients (returns to characteristics).^23^

Finally, RIF regressions were estimated at selected quantiles of the continuous birth weight distribution to assess how covariate effects vary across the distribution. Unlike mean-based models, RIF regressions estimate unconditional quantile effects, allowing identification of factors that matter more at the lower or upper tails of the distribution.

Across all multivariate analyses, confounding was addressed through simultaneous adjustments for a consistent set of maternal, child and household covariates. No causal interpretation is implied; estimated effects represent conditional associations and distributional contributions rather than causal impact. This integrated analytical approach allows both the extent and the structure of LBW inequality to be examined while maintaining coherence across binary, decomposition and distribution-sensitive methods.

### Ethical considerations

Ethical clearance for the 2018 MICS dataset was granted by the Lesotho Bureau of Statistics and UNICEF. The dataset is publicly available and fully anonymised. No direct human subjects were involved in this secondary analysis. Informed consent for data collection was obtained by MICS administrators. No identifiers are reported.

## Results

The analyses are structured to reflect the layered econometric strategy employed: from descriptive profiling, through inequality measurement, to decomposition and distributional regression. Each method addresses a distinct empirical objective, contributing to a holistic understanding of socio-economic inequality in LBW in Lesotho.

### Descriptive statistics

The final analytical sample comprises 731 children under 5 years of age with complete and valid birth weight information, drawn from the merged 2018 MICS6 Lesotho datasets. Table 1 presents summary statistics for the key variables included in the analysis. The average birth weight across the sample is approximately 3.02 kg (standard deviation [s.d.] = 0.59). Notably, 21.6% were classified as having LBW (≤ 2.5 kg), reflecting a substantial proportion of infants at risk of early-life morbidity and mortality. On average, mothers were 26 years old, and approximately 64% reported attending at least four antenatal care visits during pregnancy. A notable proportion of mothers (32%) reported not receiving a tetanus toxoid injection. Most households were located in rural areas (69%), with an average household size of five persons. Access to the Internet was reported in only 35% of households, and maternal education was skewed towards primary and secondary levels, with just 8.3% of mothers having completed higher education.

The wealth distribution, measured through the continuous wealth score and wealth quintile dummies, exhibited marked variation across the sample – a pattern that validates its inclusion in both the inequality measurement and decomposition analyses. Sanitation and energy source variables also reveal structural disparities: only 1.6% of households reported having access to a flush toilet, while 26% reported having no toilet at all. Similarly, paraffin remained the predominant household energy source, used by 62.5% of households.

These figures underscore the socio-demographic and infrastructural diversity in the Lesotho context and provide an important background for the multivariate analyses that follow.

### Association between socio-demographic factors and low birth weight

Cross-tabulation analyses and Pearson’s chi-square tests were conducted to examine the unadjusted associations between the LBW and the selected socio-demographic and household variables. These analyses are intended to provide descriptive insight into bivariate relationships and do not account for potential confounding.

Maternal functional disability was significantly associated with LBW prevalence. Children born to mothers reporting functional disability exhibited a higher rate of LBW (34.7%), compared to those whose mothers had no functional disability (19.6%) (*χ*^2^[1] = 6.40, *p* = 0.011).

Household Internet access was also significantly associated with LBW. Children from households with Internet access had a lower LBW prevalence (15.8%) relative to those without Internet (23.5%) (*χ*^2^[1] = 6.29, *p* = 0.012).

Maternal education showed a clear gradient: mothers with primary or no education had the highest LBW rates (27.9%), followed by those with secondary education (18.2%), and the lowest prevalence was among mothers with higher education (5.1%) (*χ*^2^[2] = 18.58, *p* < 0.001).

Household wealth similarly correlated with LBW outcomes. The poorest quintile had nearly double the LBW prevalence (30.0%) compared to that of the richest quintile (15.1%) (*χ*^2^[4] = 18.22, *p* = 0.001).

In contrast, no significant associations were observed for locality (urban vs rural), maternal marital status, source of drinking water, type of toilet facility or household energy source.

Adjustment for confounding and simultaneous consideration of multiple covariates are addressed in subsequent multivariate analyses, including the Fairlie and NLD decompositions and the RIF regressions.

### Distribution of birth weight and visual diagnostics

**FIGURE 1:**
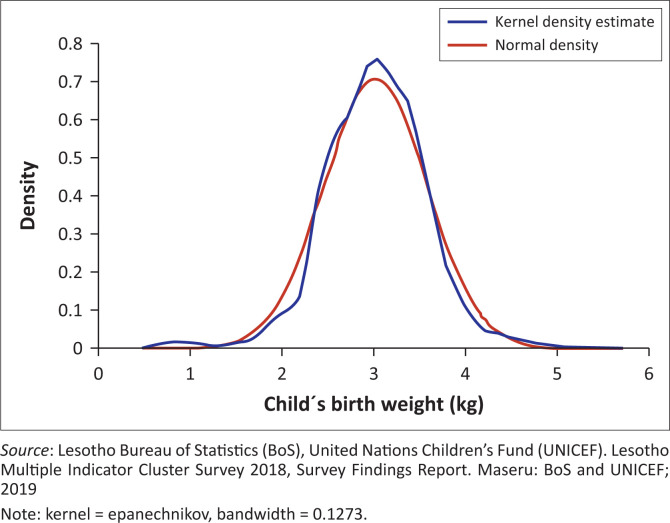
Kernel density of birth weight.

Figure 1 presents the kernel density plot of birth weight across the full sample. The distribution is positively skewed, with a pronounced spike at exactly 2.5 kg, supporting the study’s decision to define LBW as ≤ 2.5 kg. This heaping pattern has been widely observed in large-scale survey datasets and is consistent with reporting bias as a result of rounding errors.^20^

### Socio-economic inequality in low birth weight

Assessing inequality in health outcomes requires methods that capture both observed and unjust differences between population groups. Health economics provides a suite of rank-based indices for this purpose. The Concentration Index measures outcome distribution across socio-economic ranks^24^; while the EI and WI adapt CI for bounded binary outcomes such as LBW.

The three standard inequality indices, i.e., the CI, EI and the WI, were computed. Table 2 presents the results. The standard CI for LBW was estimated at −0.101, indicating a clear pro-poor inequality in LBW incidence. Both the EI (−0.084) and the WI (−0.124) corroborate this finding and correct for the bounded nature of the LBW variable. All indices are statistically significant at the 1% level, confirming that children from poorer households disproportionately suffer from LBW.

**TABLE 2:** Inequality indices for low birth weight outcomes.

Index	Estimate	s.e.	Significance
Concentration Index (CI)	−0.101	0.041	***
Erreygers Index (EI)	−0.084	0.034	***
Wagstaff Index (WI)	−0.124	0.050	***

*Source*: Lesotho Bureau of Statistics (BoS), United Nations Children’s Fund (UNICEF). Lesotho Multiple Indicator Cluster Survey 2018, Survey Findings Report. Maseru: BoS and UNICEF; 2019

Notes: The levels of statistical significance are noted as:

* *p* < 0.10;

** *p* < 0.05;

*** *p* < 0.01.

s.e., standard error.

These results, reported in Table 2, justify further decomposition to understand the drivers of the determined inequality in LBW for children under 5 years in Lesotho.

Having established the presence of significant pro-poor inequality in LBW outcomes, this subsection applies the Fairlie decomposition to quantify the relative contribution of maternal, child and household characteristics to observed disparities. Unlike linear decomposition methods, the Fairlie approach is suited to binary outcomes and allows us to examine how compositional differences across groups – such as education, ANC access and locality – account for LBW prevalence gaps between poor and rich households, and between rural and urban areas.

### Fairlie decomposition: Poor versus rich and rural versus urban

Fairlie decompositions were applied to examine the contribution of the observed characteristics to differences in LBW probabilities between two binary groupings, being the economic status (poor versus rich) and residential locality (rural versus urban). This method decomposes the difference in predicted probabilities into explained and unexplained components, based on observable covariates.

In the poor–rich comparison, 548 individuals were categorised as non-poor (reference group) and 183 as poor, based on a binary wealth classification derived from the MICS wealth index. The probability of LBW among the poor group was 32.8%, while it stood at 17.9% for the non-poor, resulting in a total disparity of 14.9% points (see Table 3). Of this, approximately 3.05% points – or around 20.5% – could be explained by observable covariates. This finding suggests that nearly 80% of the LBW inequality between the poor and the rich is attributable to the portion unexplained by the included covariates.

**TABLE 3:** Summary of Fairlie decomposition results for low birth weight inequality in Lesotho.

Comparison group	Sample (*n*)	LBW rate – Group A	LBW rate – Group B	Total gap (%)	Explained component (%)	Key significant contributors
Poor vs Rich	731 (Poor: 183; Rich: 548)	32.8% (Poor)	17.9% (Rich)	14.9	3.05 (≈ 20.5%)	−0.046** (Maternal education, *p* = 0.008); −0.014*** (ANC visits, *p* = 0.001); 0.007*** (Maternal disability, *p* = 0.008)
Rural vs Urban	731 (Rural: 504; Urban: 227)	21.6% (Rural)	21.6% (Urban)	0.04	2.7 (no significant total gap)	−0.037** (Maternal education, *p* = 0.066); −0.014** (ANC visits, *p* = 0.097); 0.008*** (Child gender [male], *p* = 0.085)

*Source*: Lesotho Bureau of Statistics (BoS), United Nations Children’s Fund (UNICEF). Lesotho Multiple Indicator Cluster Survey 2018, Survey Findings Report. Maseru: BoS and UNICEF; 2019

Notes: The explained component shows the share of the LBW probability gap because of group differences in observed characteristics. The unexplained component (not shown) captures the remainder, reflecting structural inequalities, behavioural patterns or omitted variables. Estimates are from Fairlie decomposition with 100 replications. Significance: *p < 0.10;

** *p* < 0.05;

*** *p* < 0.01.

ANC, antenatal care; LBW, low birth weight; LBW, low birth weight; vs, versus.

Among the explanatory variables, maternal education remained the most influential protective factor, accounting for −0.046 of the explained gap (*p* = 0.008). ANC visits followed, contributing −0.014 with strong statistical significance (*p* = 0.001). Maternal disability, on the other hand, contributed positively and significantly to the LBW gap (0.007, *p* = 0.008), while locality showed a modest positive contribution (0.033, *p* = 0.106), indicating a small explained component despite similar average LBW rates.

In the rural–urban decomposition, the comparison was made between 504 rural and 227 urban residents. Interestingly, the LBW probabilities were virtually identical between the two groups: 21.6% for rural and 21.6% for urban households, yielding a raw gap of only 0.04% points. Despite this negligible observed disparity, the model identified a modest explained component of 2.7% points, suggesting latent compositional differences even in the absence of an observable gap.

### Non-linear Blinder-Oaxaca decomposition

To complement the Fairlie decomposition and provide further analytical clarity on the sources of LBW disparities, the study employed a non-linear Blinder-Oaxaca decomposition based on logistic regression.^25^ This method separates the observed LBW gap into two components: the ‘characteristics and/or endowments’ effect, which captures the disparities because of group differences in covariates such as education and maternal age, and the ‘coefficients’ effect, which reflects differences in how those characteristics are rewarded or converted into outcomes across groups.

For the poor–rich comparison, the observed LBW gap was 14.9% points, consistent with the Fairlie results. As shown in Table 4, the decomposition revealed that only 2.26% points (15.1%) of this difference was attributable to differences in endowments, while the remaining 17.16% points (115.1%) stemmed from differences in coefficients – that is, how characteristics translate into LBW outcomes. This observation suggests that returns to characteristics (such as education, ANC access or disability status) differ significantly across income groups.

**TABLE 4:** Summary of non-linear Blinder-Oaxaca decomposition of low birth weight inequality.

Comparison group	Group A (*n*)	Group B (*n*)	Observed LBW gap (%)	Explained (Endowments)	Unexplained (Coefficients)
Poor vs Rich	183 (Poor)	548 (Rich)	14.90	−2.26 pp (15.1% of gap)	17.16 pp (115.1% of gap)
Rural vs Urban	504 (Rural)	227 (Urban)	0.04	7.23 pp (17609% of gap)	−7.19 pp (−17509% of gap)

*Source*: Lesotho Bureau of Statistics (BoS), United Nations Children’s Fund (UNICEF). Lesotho Multiple Indicator Cluster Survey 2018, Survey Findings Report. Maseru: BoS and UNICEF; 2019

Notes: Group A is the comparison group (Poor or Rural); Group B is the reference group (Rich or Urban). Explained (characteristics) are differences in average covariates; Unexplained (coefficients) are differences in marginal effects of covariates. Values in percentage points (pp). Percentages > 100% or negative signs indicate overcompensation or countervailing effects.

LBW, low birth weight; vs, versus.

The magnitude of the coefficient effect implies that even if poor mothers had the same average characteristics as rich mothers, structural or behavioural disparities in the health system – including the quality, reach or responsiveness of services – would still sustain most of the LBW inequality. The magnitude of the coefficient effect thus indicates that most of the LBW gap persists even after accounting for the differences in observed characteristics between poor and rich mothers.

In contrast, the rural–urban decomposition yielded a negligible raw LBW gap of 0.04% points, again aligning with the Fairlie findings. However, the non-linear decomposition estimated a characteristics effect of 7.23% points and a coefficients effect of −7.19% points, which nearly offset one another. RIF regressions were estimated to further examine the distributional nature of these disparities.

### Recentred influence function regression results

The RIF regression developed by Firpo, Fortin and Lemieux^19^ allows for estimating covariate effects across the outcome distribution. This estimation is essential for capturing tail vulnerabilities and understanding whether covariate effects are stronger among babies with very low (i.e., 25th percentile) or relatively high birth weights (i.e., 75th percentile).

RIF regressions were estimated at the 28th, 50th (median) and 75th percentiles of the birth weight distribution. The RIF regression results (Table 5) show that the effects of maternal and household characteristics vary across the distribution. However, many covariates lack statistical precision at the lower and median quantiles, and only a few reach significance at the upper end. This distributional instability is not uncommon in survey-based RIF analysis and reflects both sample variability and potential non-linear interactions.

**TABLE 5:** Recentred influence function regression results at 28th, 50th and 75th quantiles.

Variables	Q (0.28)	Q (0.50)	Q (0.75)
Antenatal care visits	−0.015 (0.013)	−0.005 (0.014)	0.027 (0.057)
Tetanus toxoid injections	−0.132 (0.243)	−0.162 (0.221)	−0.146 (0.224)
Maternal disability (1 = No disability, 0 = Otherwise)	0.223 (0.181)	0.318 (0.203)	0.451 (0.236)*
Maternal marital status	0.061 (0.047)	0.084 (0.052)	0.112 (0.057)*
Maternal age	−0.007 (0.017)	0.005 (0.019)	0.031 (0.026)
Maternal education	0.037 (0.159)	−0.002 (0.148)	0.244 (0.229)
Child gender (1 = Female, 0 = Male)	0.386 (0.274)	0.517 (0.299)*	0.204 (0.373)
Household size	−0.040 (0.042)	−0.012 (0.044)	0.045 (0.060)
Internet access	0.194 (0.186)	0.401 (0.282)	0.503 (0.299)*
Toilet type	0.481 (0.445)	0.512 (0.406)	0.088 (0.529)
Energy source	0.305 (0.297)	0.018 (0.366)	0.123 (0.380)
Water source	−0.156 (0.233)	−0.104 (0.211)	−0.411 (0.479)
Continuous wealth score	−0.041 (0.226)	0.098 (0.243)	−0.067 (0.276)
Locality (1 = Rural, 0 = Urban)	−0.198 (0.217)	0.153 (0.382)	0.146 (0.398)
*Constant*	−0.133 (0.942)	−0.507 (1.094)	−0.619 (1.267)

*Source*: Lesotho Bureau of Statistics (BoS), United Nations Children’s Fund (UNICEF). Lesotho Multiple Indicator Cluster Survey 2018, Survey Findings Report. Maseru: BoS and UNICEF; 2019

Notes: Standard errors in parentheses. Significance levels:

* *p* < 0.10;

** *p* < 0.05;

*** *p* < 0.01.

Q(*) indicates the distribution quintile of continuous birth weight.

At the 28th percentile, no variable was statistically significant at conventional thresholds, although some directional effects are noteworthy. For example, maternal disability was associated with a positive coefficient (0.223), indicating that children of mothers without functional disabilities tended to have higher birth weights in this vulnerable zone. Similarly, child gender (female) and Internet access showed moderately positive associations with birth weight, although not statistically distinguishable from zero. Notably, locality had a negative but insignificant coefficient (−0.198), suggesting a slightly higher risk of LBW among rural dwellers at the lower end of the distribution.

At the median (50th percentile), a somewhat clearer pattern emerged. Child gender (female = 1) became statistically significant (*β*= 0.517, *p* < 0.10), suggesting that girls were more likely to achieve median-level birth weights, reinforcing biological or care-related gender differentials. Maternal marital status (*β* = 0.084) and disability status (*β* = 0.318) showed positive effects with marginal significance. Internet access also gained relevance (*β* = 0.401), although not significant at 10%. These results imply that both structural constraints (e.g., disability) and household-level assets (e.g., digital connectivity) begin to matter more around the median.

At the 75th percentile, the effects of socio-economic and maternal characteristics were strongest. Maternal disability again showed a sizeable positive association with birth weight (*β* = 0.451, *p* = 0.056), while marital status also became borderline significant (*β* = 0.112, *p* = 0.051), suggesting that formal unions may offer social or financial stability beneficial to late-gestation development. Importantly, Internet access had a large and near-significant effect (*β* = 0.503, *p* = 0.093), likely reflecting improved access to health information or support networks. However, the locality variable showed no significant effect at any point along the distribution, although the direction of the variable remained positive at the upper tail.

Finally, the continuous wealth index was not statistically significant at any quantile, and the sign of the index varied across the distribution – slightly negative at the 75th percentile. This finding suggests that household economic status, while important for access, may be mediated by other structural or behavioural variables, particularly with respect to birth outcomes in Lesotho.

Overall, the RIF regressions underscore the importance of examining health inequality across the full birth weight distribution, rather than focusing solely on binary or mean outcomes. The regressions reveal that maternal disability, marital status, child gender and digital connectivity are more relevant in shaping outcomes at the upper tail, while education and ANC visits may play a modest protective role towards the lower end, albeit with limited precision. The inclusion of locality added minimal explanatory power after accounting for other socio-demographic controls, suggesting that geographic disparities may be mediated by infrastructure and health-seeking variables rather than by direct location effects. These findings support the application of RIF methods as a valuable complement to binary decomposition approaches, offering richer insights for targeting equity-oriented maternal and neonatal health interventions.

## Discussion

This study examined socio-economic inequalities in LBW outcomes in Lesotho using a hybrid econometric framework that combined rank-dependent inequality measures, decomposition techniques and distribution-sensitive regressions. The findings provide clear evidence that LBW in Lesotho is unequally distributed, disproportionately affecting children from poorer households. Importantly, the magnitude and structure of this inequality vary across socio-economic groups and across the birth weight distribution, highlighting that LBW disadvantage is both systematic and heterogeneous.

Across methods, three principal findings emerge. Firstly, LBW is significantly concentrated among poorer households, as confirmed by the Concentration Index and its corrected variants, indicating a persistent wealth gradient in early-life health outcomes. Secondly, decomposition analyses reveal that only a modest share of the poor–rich LBW gap is explained by observable maternal and household characteristics, such as education and ANC utilisation. The majority of the disparity remains unexplained, pointing to structural and contextual factors that influence how socio-economic characteristics translate into birth outcomes. Thirdly, distribution-sensitive RIF regressions show that different determinants matter at different points of the birth weight distribution: maternal education and ANC are more relevant among children at the lower tail, while maternal disability, marital status and digital connectivity exert stronger influence at the upper tail.

Taken together, these findings suggest that LBW inequality in Lesotho reflects not only compositional disadvantage but also unequal returns to health-promoting characteristics. From a public health perspective, this result implies that expanding access to services alone may be insufficient to close LBW gaps if the equality, responsiveness and inclusiveness of the maternal health system remain uneven. The prominence of the unexplained component in the poor–rich decomposition underscores the potential role of health system factors, environmental risks and social exclusion in shaping neonatal outcomes, particularly among socio-economically disadvantaged mothers.

In what follows, the main results from each method are interpreted in detail and contextualised within the existing literature.

### Socio-economic inequality in low birth weight: Concentration Index, Erreygers Index and Wagstaff Index

The presence of socio-economic inequality in LBW was confirmed by using three standard rank-dependent indices. The CI = (−0.108), EI = (−0.084) and WI = (−0.095) were all negative and statistically significant, indicating a pro-poor concentration of LBW. Taken together, these wealth differentials underscore persistent socio-economic disparities in maternal and child health.

These findings are in line with regional evidence from Fotso^14^ and Elsey et al.,^11^ who observed similar inequalities in child nutrition and neonatal outcomes across SSA. The magnitude of these indices is also consistent with global health inequality literature,^15^ reinforcing the need for redistributive and equity-focused maternal and child health interventions.

### Logit model: Baseline covariate effects

The baseline logistic regression revealed key correlates of LBW in Lesotho. ANC attendance and maternal education emerged as strong protective factors, aligning with findings from O’Donnell et al.^24^ and Black et al.^2^ Maternal disability significantly increased the odds of LBW, highlighting the compounded vulnerabilities faced by mothers with functional impairments. This finding highlights the vulnerability of children born to mothers facing physical or health challenges that may affect pregnancy outcomes.

Importantly, the inclusion of residential locality showed that urban mothers were significantly less likely to report LBW outcomes, suggesting a protective effect linked to location-specific infrastructure, services or living conditions. These results highlight the relevance of both human capital and structural access in influencing maternal and neonatal health outcomes in Lesotho. These null findings suggest that, in this context, maternal education, functional disability, Internet access and household wealth are more salient correlates of LBW than basic environmental or infrastructural characteristics are.

### Fairlie decomposition: Poor–rich and rural–urban disparities

The updated Fairlie decomposition showed that the LBW gap between poor and rich households (14.9% points) was only modestly explained by observable characteristics (20.5%). Key contributors included maternal education (−0.046, *p* < 0.01), ANC visits (−0.014, *p* < 0.01) and maternal disability (0.007, *p* < 0.01). Specifically, the latter contribution suggests that children born to mothers with disabilities are more likely to have children with LBW than their counterparts without functional disabilities are – possibly as a result of reduced care access, stigma or health vulnerability.

Other variables such as child gender, maternal age, infrastructure access (water, sanitation, and energy), Internet access and wealth status itself were found to be insignificant in explaining the gap in LBW prevalence in Lesotho. The residential locality contributed positively to the explained component, although not significantly, suggesting latent structural gaps between rural and urban environments.

For the rural–urban comparison, although the overall LBW rates were identical (21.6%), the decomposition uncovered latent compositional differences (explained gap = 2.7%). Again, education and ANC use featured as protective factors, while child gender (male) had a marginally positive contribution. Despite the negligible surface-level disparity, these results signal possible hidden vulnerabilities in rural settings, which may emerge under strain. The findings echo O’Donnell et al.^9^ by illustrating how inequalities often stem more from how characteristics convert into outcomes than their mere presence.

In this context, maternal education again showed a near-significant negative contribution (−0.037, *p* = 0.066), showing a negative association between maternal education and LBW prevalence. This result confirms the well-documented protective role of maternal education in improving neonatal outcomes. ANC visits also contributed negatively (−0.014, *p* = 0.097), showing that ANC visits contributed negatively to the explained rural–urban gap. A small positive contribution from child gender (0.008, *p* = 0.085) was observed, showing a small positive effect from the male child gender on the explained component.

Other variables – including household size, access to toilet facilities, energy source and Internet connectivity – had statistically insignificant but directionally varied effects. Notably, maternal disability did not significantly contribute to rural–urban differences, showing no significant contribution of maternal disability to rural–urban differences.

Furthermore, the Fairlie decompositions illustrate that in Lesotho, inequalities in maternal education, health-seeking behaviour (ANC) and disability status are important compositional factors influencing LBW disparities – especially along economic lines. However, the large unexplained component indicates that a substantial share of LBW inequality remains unaccounted for by observed covariates.

In sum, taken together, the decomposition results suggest that differences in maternal education, ANC utilisation and disability status account for part of the observed wealth gradient, while rural–urban differences are limited once compositional factors are considered. Thus, these findings highlight the importance of further investigation into unobserved or contextual factors – such as service quality, environmental exposure or behavioural risks – that may possibly drive residual LBW inequality.

#### Non-linear Blinder-Oaxaca decomposition insights

The NLD results reinforced the Fairlie findings and added structural insight. For the poor–rich comparison, the endowment effect was −2.26% points (15.1%), while the coefficient effect was 17.16% points (115.1%). This result strongly suggests that even when poor mothers have similar characteristics to their wealthier counterparts, the returns to those characteristics are structurally diminished. These diminished returns likely reflect differential service quality, health system responsiveness or deeper social exclusion, corroborating decomposition-based analyses of LBW and health inequality in SSA^15,26,27^

In the rural–urban model, a counterbalancing dynamic was found: a positive endowment effect (7.23 *pp*) was offset by an equally strong negative coefficient effect (−7.19 *pp*), leading to a net-zero difference. This dynamic resulted in a net-zero predicted difference in LBW between urban and rural residents. While this outcome may suggest surface-level parity in outcomes, the opposing directions of the decomposition components indicate latent compensatory dynamics: rural mothers may be disadvantaged in characteristics (e.g., lower education and poorer service access), but disadvantage is counterbalanced by slightly more favourable or at least neutral returns to those characteristics. This finding could reflect more uniform public health delivery in rural areas, or differential patterns of care-seeking or reporting in urban contexts.

Taken together, the NLD results add depth to the Fairlie findings. For the poor–rich comparison, the results confirm that the LBW inequality is predominantly structural, driven by the fact that poor mothers receive weaker marginal health returns from similar traits. In contrast, rural–urban disparities appear statistically negligible, although latent differences in characteristics and their translation into outcomes do exist and operate in opposite directions.

These findings reinforce the value of a hybrid analytical framework – combining binary decompositions (Fairlie and NLD) with distribution-sensitive methods (RIF) – to capture both compositional and structural insights into health inequality. This reinforcement suggests that urban compositional advantages (e.g., education and service access) are neutralised by relatively weaker returns, potentially because of overcrowded health systems, reporting bias or urban poverty traps. Conversely, rural mothers may experience more consistent returns from fewer inputs, possibly as a result of more uniform service delivery or targeted rural programmes.

### Recentred influence function regression: Distribution-sensitive covariate effects

RIF regressions offered further insights by estimating covariate effects at multiple points along the birth weight distribution, which confirmed the utility of distribution-sensitive methods. More importantly, coefficients are interpreted as changes in the conditional quantile of LBW probability because of one-unit changes in covariates.

At the 28th percentile, which captures children most at risk of LBW, maternal disability and child gender (female) had moderately positive but statistically insignificant effects. These results suggest that basic maternal health status and infant sex may influence outcomes in vulnerable strata, although with imprecise estimation.

At the median, female gender became significant (*β* = 0.517, *p* < 0.10), while maternal disability and marital status showed increased positive effects. Internet access also gained influence, highlighting the intersection of structural and informational resources. These findings support the idea that digital connectivity and household stability matter for mainstream birth outcomes.

At the 75th percentile, the most advantaged segment of the birth weight distribution, the effects were strongest: maternal disability (*β* = 0.451, *p* = 0.056), marital status (*β* = 0.112, *p* = 0.051) and Internet access (*β* = 0.503, *p* = 0.093) all approached or reached statistical significance. With regard to Internet access, this pattern may reflect broader socio-economic advantages or access to health information influencing maternal and child health outcomes.

However, jointly, these results suggest that structural assets and social capital (e.g., being married, digitally connected or non-disabled) exert cumulative protective effects at the upper end of the birth weight spectrum. Importantly, residential locality (urban vs rural) had no statistically significant effect at any point along the distribution, despite entering all models. This outcome implies that location per se may be less important than the covariates it proxies for, such as access, education and health behaviour.

The decision to model the 28th percentile, rather than a conventional quartile, was based on evidence of heaping at 2.5 kg, a common issue in birth weight data from low- and middle-income countries.^28^ This decision was justified by using the 28th percentile to represent the low-weight threshold more accurately, thus improving the interpretive clarity of distributional effects.

Collectively, the RIF results highlight that different drivers operate at different points of the birth weight distribution. Lower tail outcomes are shaped more by maternal education and health inputs, while upper tail outcomes reflect cumulative socio-structural advantages. This layered insight justifies the incorporation of RIF techniques in future health equity diagnostics.

Although several estimates approached but did not reach conventional significance thresholds, the magnitude and consistency of effects – particularly for maternal disability (*β* ≈ 0.45 at the 75th percentile) and Internet access (*β* ≈ 0.50) – indicate practically meaningful associations. Given the limited sample size, these moderate coefficients suggest effect sizes large enough to influence birth outcomes even if statistical precision is limited. Thus, the results should be interpreted in terms of substantive importance rather than p-values alone.

### Model validity and diagnostic strength

The robustness of the models was confirmed through multiple diagnostic tests. The logistic regression model yielded a *pseudo R*^2^ of 0.0662 – modest but typical for cross-sectional health data. The RIF-mean model showed an adjusted *R*^2^ of 0.0451, and the RIF-quantile models had low but acceptable *R*^2^ values, consistent with expectations for influence-function regressions.

The Hosmer–Lemeshow goodness-of-fit test for the logistic model was non-significant (*p* > 0.05), indicating that predicted probabilities closely matched observed outcomes. Multicollinearity checks using variance inflation factors showed no concern, and residual diagnostics revealed no heteroscedasticity. These findings support the internal validity and statistical soundness of the estimations.

## Conclusion and policy recommendations

This study set out to examine socio-economic inequalities in LBW outcomes in Lesotho, using a hybrid econometric strategy that combined simulation-based Fairlie decompositions, NLD and RIF regressions. The findings provide robust evidence that LBW is unequally distributed, disproportionately affecting children born into poorer households, and that both compositional and structural inequalities underpin these disparities. While rural–urban differences appeared small at the aggregate level, latent disparities were nevertheless evident in how health-promoting factors translated into outcomes across localities.

Importantly, the study found that structural advantages – such as maternal education, ANC attendance and digital connectivity – were not only unevenly distributed but also yielded differential effectiveness across socio-economic contexts. The RIF regressions revealed that the influence of these variables varies by position in the birth weight distribution: education and ANC visits had their strongest effects at the 28th percentile, at which children are most vulnerable to severe LBW. By contrast, maternal disability status, Internet access and marital status became more influential at the 75th percentile, pointing to the cumulative protective value of social and structural resources.

These findings reinforce the importance of moving beyond mean-based assessments and instead embracing approaches that diagnose both observable disadvantage and structural asymmetries in returns to health-promoting factors. In doing so, the article contributes empirical support for Lesotho’s pursuit of SDG 3 (Good Health and Well-being) and SDG 10 (Reduced Inequalities), particularly in the context of maternal and neonatal outcomes.

### Limitations of the study

While the hybrid analytical framework improves the robustness of findings, several limitations should be noted:

- Omitted Clinical Factors: The study used the 2018 Lesotho MICS dataset, which either partially or fully lacks data for key biomedical covariates (e.g., gestational age, anaemia, hypertensive disorders and substance use). This shortcoming limits the model’s ability to control for clinical determinants of LBW and introduces potential omitted variable bias.
- Data Exclusion and Sample Loss: As a result of missing or inconsistent responses – especially regarding birth weight – some observations were excluded for data integrity. If the missingness is non-random, selection bias may arise. Although a complete-case strategy may have reduced some statistical power, but sensitivity checks indicated that key associations remained robust, with no sign reversal or any large coefficient shift. This robustness supports the stability of the estimated relationships.
- Cross-sectional Design: All analyses are based on cross-sectional data, which limits causal inference. While decompositions and RIF methods offer powerful diagnostic insights, they cannot confirm temporality or causality.
- Explained Gap Significance in Fairlie Decomposition: Although the explained portion of the poor–rich Fairlie decomposition was estimated at 3.05% points (≈ 20.5%), its statistical significance as a whole could not be tested directly because of limitations in Fairlie’s simulation-based approach. However, individual covariates such as maternal education (*p* = 0.008), ANC visits (*p* = 0.001) and maternal disability (*p* = 0.008) were statistically significant.

### Policy recommendations

Given the hybrid analytical design combining decomposition and distributional regression methods, the results reveal multi-level drivers of LBW inequality in Lesotho – spanning structural, behavioural and compositional factors. Consequently, the recommendations are framed at a systems level rather than tied to single covariates, reflecting the integrated nature of inequality in health outcomes. Based on these findings, six key recommendations are proposed:

1. *Invest in Maternal Education:* Female education is a consistent protective factor across decomposition and RIF models. It enhances health literacy, health-seeking behaviour and decision-making. Policies should prioritise secondary school completion for girls and consider embedding maternal health literacy into curricula.
2. *Expand and Standardise ANC Coverage:* Beyond increasing ANC uptake, the focus should be on quality and responsiveness, especially in under-resourced rural facilities. Evidence-based, respectful maternity care should be institutionalised to ensure that access translates into outcomes.
3. *Close Structural Gaps in Rural Areas:* Although locality had no significant direct effect in RIF models, its role in the logistic model suggests embedded structural disadvantages. Investments in water, sanitation, energy, transport and digital infrastructure should be synchronised with health strategies, supported by mobile outreach models.
4. *Target Vulnerability Across the Distribution:* Given that different socio-economic drivers are relevant at different parts of the birth weight distribution, interventions must be distribution-sensitive. For instance, ANC and maternal education are more critical at the lower end, while Internet access and marital stability gain influence at higher percentiles.
5. *Strengthen Health Information Systems:* Lesotho should build its national capacity to collect biomedical and behavioural health data to improve future targeting. Integration of MICS, DHS and health facility records could enrich data quality and allow for better programme design.
6. *Layer Social Protection with Health Interventions:* Consider conditional cash transfers, transport vouchers or maternal nutrition support to reduce economic vulnerability during pregnancy. These approaches complement existing health services and are supported by global evidence (e.g.,^16^) on demand-side financing and improved neonatal outcomes.

This study demonstrates that socio-economic inequality in LBW is both real and distributionally complex in Lesotho. The study reveals that inequality stems not only from who mothers are (i.e., endowments) but also from how the health and social systems treat those endowments. With deliberate, equity-focused action, it is possible to reduce LBW disparities and ensure that all children – regardless of background – have an equal start to life.
